# A Rootstock Provides Water Conservation for a Grafted Commercial Tomato (*Solanum lycopersicum* L.) Line in Response to Mild-Drought Conditions: A Focus on Vegetative Growth and Photosynthetic Parameters

**DOI:** 10.1371/journal.pone.0115380

**Published:** 2014-12-22

**Authors:** Erik T. Nilsen, Joshua Freeman, Ruth Grene, James Tokuhisa

**Affiliations:** 1 Department of Biological Sciences, Virginia Tech, Blacksburg, Virginia, United States of America; 2 North Florida Research and Education Center, University of Florida, Quincy, Florida, United States of America; 3 Department of Plant Pathology, Physiology and Weed Science, Virginia Tech, Blacksburg, Virginia, United States of America; Chinese Academy of Sciences, China

## Abstract

The development of water stress resistant lines of commercial tomato by breeding or genetic engineering is possible, but will take considerable time before commercial varieties are available for production. However, grafting commercial tomato lines on drought resistant rootstock may produce drought tolerant commercial tomato lines much more rapidly. Due to changing climates and the need for commercial production of vegetables in low quality fields there is an urgent need for stress tolerant commercial lines of vegetables such as tomato. In previous observations we identified a scion root stock combination (‘BHN 602’ scion grafted onto ‘Jjak Kkung’ rootstock hereafter identified as 602/Jjak) that had a qualitative drought-tolerance phenotype when compared to the non-grafted line. Based on this initial observation, we studied photosynthesis and vegetative above-ground growth during mild-drought for the 602/Jjak compared with another scion-rootstock combination (‘BHN 602’ scion grafted onto ‘Cheong Gang’ rootstock hereafter identified as 602/Cheong) and a non-grafted control. Overall above ground vegetative growth was significantly lower for 602/Jjak in comparison to the other plant lines. Moreover, water potential reduction in response to mild drought was significantly less for 602/Jjak, yet stomatal conductance of all plant-lines were equally inhibited by mild-drought. Light saturated photosynthesis of 602/Jjak was less affected by low water potential than the other two lines as was the % reduction in mesophyll conductance. Therefore, the Jjak Kkung rootstock caused aboveground growth reduction, water conservation and increased photosynthetic tolerance of mild drought. These data show that different rootstocks can change the photosynthetic responses to drought of a high yielding, commercial tomato line. Also, this rapid discovery of one scion-rootstock combination that provided mild-drought tolerance suggests that screening more scion-rootstock combination for stress tolerance may rapidly yield commercially viable, stress tolerant lines of tomato.

## Introduction

Tomato production is an important aspect of the agricultural economy in the US and other nations. In Virginia, the fresh market tomato industry ranks 5^th^ in the United States, and had a value of 51 million dollars in 2010 (USDA-NASS, 2010). Weather extremes, insect and disease pests annually challenge tomato fruit productivity and these factors are amplified by global climate change and new regulations on soil fumigant use. For instance, abiotic-stresses such as drought and salinity can cause a major loss for tomato production [1]–[3]. Moreover, the potential for drought-induced reduction in productivity is increasing due to a changing climate and consequent movement of tomato production into less arable locations [1], [4], [5]. To maintain commercial viability, producers must be equipped with stress tolerant cultivars that maximize production and promote sustainable practices. Classically, breeders have developed new cultivars that have higher photosynthesis and productivity through introgression of the advantageous trait by multiple crosses. The complicated genetic control of traits like photosynthetic response to drought may require years if not decades of breeding for the development of a commercially acceptable cultivar. Having a rapid method to introduce desirable photosynthetic responses to stress for crops would be a tremendous benefit for agriculture in the U.S.

Searches for genes that have the potential to increase resistance to abiotic stress in tomato have yielded several candidates. For example, when the gene for *CaXTH3* (xyloglucan endotransglucosylase/hydrolase) from hot pepper was constitutively expressed in lines of tomato, tolerance to water stress was enhanced [6]. A gene (*SpERD15*) from *Solanum pennellii* Correll, when expressed into tobacco, resulted in increased drought resistance in the transgenic plants [7]. Although these and other candidate genes have been identified that may increase tomato productivity during water stress, an assemblage of interacting genes are also known to be controlled by global regulators of stress tolerance in tomato [8]. Therefore, utilizing genetic engineering to improve commercial tomato photosynthesis under water stress conditions has potential, but will take time to result in commercially viable, stress resistant lines.

Alternatively, new lines of commercial tomatoes with water stress resistant photosynthesis could be rapidly developed by utilizing grafting technologies. Grafting scions of commercial cultivars onto water stress resistant rootstock has been suggested as a viable option for engineering resistance to biotic and abiotic stresses in crops such as tomato [4], [9] and has been practiced for decades in Asian countries [10]. This approach with drought resistant rootstocks has been successful in several tree crops [11]–[13], shrub crops [14], [15] and vines [16], [17]. However, research on the value of rootstocks to drought resistance in vegetable production has lagged behind [4].

Currently, wild tomato and other plant lines have not been phenotypically characterized as rootstocks, especially under the types of conditions encountered in the field. Our long-term goal is to identify, characterize and introduce grafted tomato plants with trait combinations that are responsive to regional growing conditions and new cultural practices, which will maximize photosynthesis and minimize adverse environmental impacts. Grafting existing cultivars onto rootstocks of wild relatives may restore various stress resistances present in wild relatives while retaining the hard-won traits of domestication. In previous research, we identified one scion-rootstock combination that had a qualitative drought-tolerance phenotype when compared to non-grafted plants. This was observed in seedlings of ‘BHN 602’ scion grafted onto ‘Jjak Kkung’ rootstock in a growth chamber setting. Here we report on vegetative growth parameters and photosynthetic characterization, during mild-drought, of BHN602/Jjak Kkung scion-rootstock graft combinations in comparison with BHN602 grafted to a different rootstock and a non-grafted control. Our goal was to characterize the photosynthetic characteristics of scion-rootstock combinations in relation to vegetative growth characteristics and search for possible physiological factors associated with the mild-drought tolerance of the BHN602/Jjak Kkung scion-rootstock combination.

Based on our previous research, we predicted that the effects of mild-drought on vegetative growth characteristics of grafted plant lines would be less than that for the non-grafted control. In particular, we hypothesized that during mild-drought, stomatal conductance and photosynthesis would decrease less for the BHN602/Jjak Kkung scion-rootstock combination than the other grafted plants or the non-grafted control. Moreover, we proposed that the effect of mild-drought on photosynthesis would be primarily due to reductions in stomatal conductance, but not reductions in mesophyll conductance for all grafted and non-grafted plants because we used a mild-drought treatment. In addition, we hypothesized that the regression between photosynthesis and leaf water potential will be less steep for the BHN602/Jjak Kkung scion-rootstock combination plants in comparison with that of other grafted plants and the non-grafted control.

## Materials and Methods

### Rootstocks and scion variety

The scion used for all grafted and non-grafted treatments was the determinant cultivar ‘BHN 602’ (BHN Seed, Immokalee, FL). Scions were grafted onto either of two different rootstocks, ‘Cheong Gang’ or ‘Jjak Kkung’ (Seminis Vegetable Seeds, St. Louis, MO), utilizing a modified Japanese tube graft at the two-leaf stage [18]. Therefore, we used three different plant lines: 1); BHN 602 non-grafted (hereafter identified as 602), 2); BHN 602 scion grafted onto Cheong Gang rootstock (hereafter identified as 602/Cheong), and 3); BHN 602 scion grafted onto Jjak Kkung rootstock (hereafter identified as 602/Jjak). Grafting the scion above the rootstock cotyledons is known to result in extensive lateral branch outgrowths of the rootstock [19]. Therefore, in order to minimize any influence of above ground growth of the rootstock, which would interfere with our analysis of scion drought tolerance, all grafts were made below the rootstock cotyledons. Soil-less medium was used for the production of all transplants. After grafting was performed, seedlings were placed in a high humidity chamber with controlled temperature to heal the graft union [20]. After one week, seedlings were removed from the chamber and placed in a greenhouse for 10–14 days before transplanting. Since the grafting was made below the rootstock cotyledon, care was taken at planting to maintain the graft union above the soil line to avoid adventitious root formation by the scion. Plants were transplanted into 4-inch pots in a commercial growth media (Sungro Metro-Mix 300 series; Sun Gro Horticulture Canada Ltd., BC, Canada).

### Experimental design

Seeds were germinated and plants were grafted at Virginia Tech Eastern Shore AREC, in Painter Virginia. After grafted and non-grafted transplants reached 20 cm in height they were shipped to the Virginia Tech Blacksburg campus for exposure to mild drought. Mild-drought was defined as a water withholding period that induced wilting, but no permanent damage to the leaf lamina. At the Virginia Tech Biology/Virginia Bioinformatics Institute Plant Growth facility, 20 plants of each line were transplanted into commercial growth media (Pro-mix BX with Biocide; Wetsel Greenhouse Supply; Harrisonburg, VA, USA) in 5 gallon plastic pots. Each plant was numbered consecutively between 1 and 60 and an equal number of each line was randomly assigned to the control and mild-drought treatment resulting in a sample size of 10 plants per line per treatment. All plants were watered daily, to full pot saturation at mid morning, until water was withheld from the mild-drought treatment plants. Plants were rotated, within the bench, every two days to minimize any location effect in the greenhouse. Fertilizer (10N:10P:10K plus micronutrients) was applied at a rate of 12 g pot^−1^ week^−1^ throughout the experiment. Leaf greenness (Minolta chlorophyll meter model SPAD502) was measured weekly on the first fully mature leaf on each plant to verify that no nutrient limitations occurred through the experiment. All flower buds were pinched off before development so that variability in fruit yield would not influence our vegetative growth and photosynthesis measurements.

At 19 days after transplanting, water was withheld from the mild-drought treatment plants, while the control plants continued to be watered daily. The mild-drought treatment continued for one additional day past the day at which the mean water potential induced full stomatal closure at midday. Mild-drought treatment plants were re-watered on day 29 after transplanting. Therefore, the mild-drought treatment constituted withholding water for 10 days.

Growth was measured for all plants throughout the experimental period by determining main axis length, total shoot length, number of lateral shoots and number of leaves. Three days after re-watering the drought-stressed treatment (day 32 after transplant), all plants were harvested for analysis of final growth and allocation patterns. At final harvest above ground material was removed, separated into main axis stem, main axis leaves, lateral shoot stem and lateral shoot leaves. All leaves were separated into leaf-lamina and leaf rachis. A subsample of leaflets was separated to determine the leaf-lamina specific leaf weight for each plant. The leaf area of the leaf-lamina subsample was measured (LICOR leaf area meter model 3100). All plant materials were dried in a forced air oven at 60°C to constant dry weight. The leaf-lamina, specific leaf area (cm^−2^/g dry weight) was multiplied by the total leaf-lamina dry weight (g) values to determine leaf-lamina area per plant. An estimated relative growth rate (RGR) was calculated as the estimated change in weight during each growth time period divided by the estimated weight at the initial time step for that increment for all plants individually. Plant weight was estimated by multiplying stem length (measured for each plant at each time step) times total weight per stem length (measured for each plant at the end of the experiment). Relative growth rate ranged between 0.56 and 0.66 g day^−1^ g^−1^ for all lines and treatments before the mild-drought treatment was initiated.

A weather station was established at the height of the tomato canopy in the growth room. A data logger (Campbell Scientific, model 21x) recorded air temperature and relative humidity (Campbell Scientific, Temp/RH sensor model 517) and photosynthetic photon flux density (PPFD) (Licor Biosciences, PAR sensor model 190s). Data was logged each second and the mean for each 10-minute period was saved to describe the meteorological conditions for the experiment.

### Physiological measurements

Midday leaf water potential and midday stomatal conductance were monitored on three randomly selected plants of each line and treatment daily from day 5 and 15 respectively after transplanting to the end of the experiment (day 31). Beginning on day 23 light saturated photosynthesis (A_max_), and CO_2_ saturated photosynthesis (A_sat_) were measured for three randomly selected plants for each line and treatment. Measurements were made between 10:00AM and 5:00PM to make sure radiation on the whole plant was at or near light saturation. Plants were measured in pairs, one plant of each treatment of each plant line during each time bracket (late morning, midday, and early afternoon) to minimize any time related bias in the data. Initially light response curves and CO_2_ response curves were performed (LICOR Biosciences gas exchange system model 6400xt) to determine appropriate light intensity and CO_2_ concentration for saturation (data not presented). A five-minute auto-log program was used to determine light saturated photosynthesis (A_max_) at 400 ppm CO_2_, 25°C air temperature, 1500 µmol m^−2^ s^−1^ PPFD, and 70–80% relative humidity. Following the A_max_ determination, CO_2_ concentration was raised to 1400 ppm. After an equilibration period of 10 minutes a 5-minute auto-log program was run at 1400 ppm CO_2_, 25°C air temperature, 1500 µmol m^−2^ s^−1^ PPFD, and 70–80% relative humidity to determine the CO_2_ saturated rate of photosynthesis (A_sat_). While the auto-log programs were running, stomatal conductance was measured on three additional recently matured leaves (Licor Biosciences porometer, model 1600) and dark adapted Fv/Fm was measured on three additional mature leaflets (Optisciences fluorometer model OS500). Recently mature leaves were considered the first leaves on the branch that had attained full size. Dark-adaptation clips were installed on the selected leaflets before the auto-log program began so that the leaflets would be dark adapted for greater than 15 minutes before Fv/Fm determinations.

## Results

### Growth conditions

**Figure 1:**
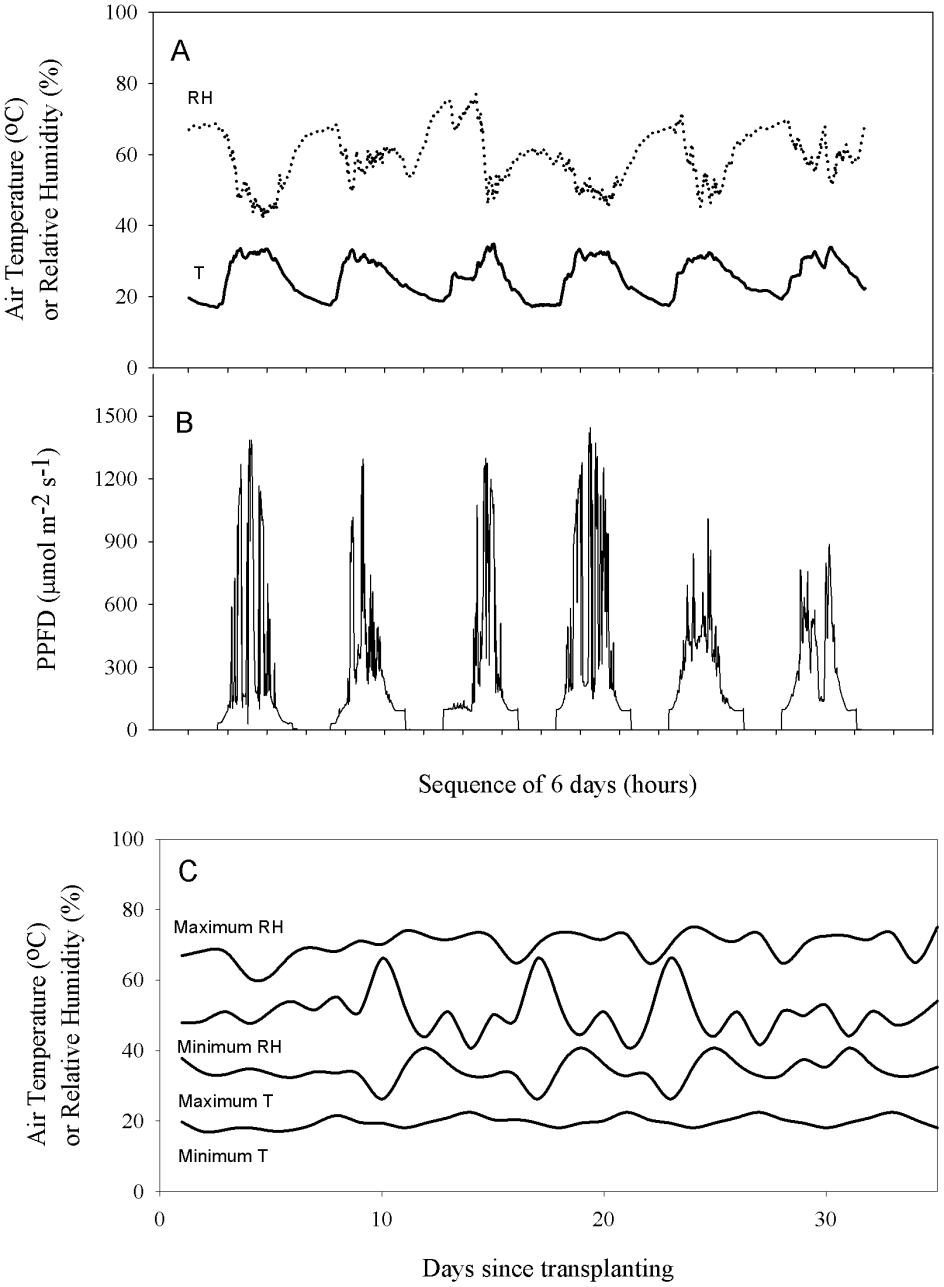
The microclimatic conditions at canopy height in the growth room used for an experiment on grafted and non-grafted lines of tomato are shown. A) Six representative diurnal cycles of air temperature and relative humidity; B) Six representative diurnal cycles of photosynthetic photon flux density (PPFD); C) Daily maximum and minimum air temperature and relative humidity over the 30 day experimental period.

On each day of the experiment, minimum temperature and maximum relative humidity occurred just before dawn, and air temperature increased to a maximum near midday when minimum relative humidity occurred (Fig. 1A). After 2:00PM temperature gradually decreased and relative humidity gradually increased until just before dawn. On sunny days maximum PPFD reached 1500 µmol m^−2^ s^−1^. However, PPFD varied significantly among days and within days (Fig. 1B). Maximum daily relative humidity varied from 75% to 65% over the experimental growth period and minimum daily relative humidity varied from 42% to 64% (Fig. 1C). Daily maximum air temperature varied from 41°C to 28°C among days during the growth period and daily minimum air temperature varied from 18°C to 22°C (Fig. 1C).

### Vegetative plant growth above ground

**Figure 2:**
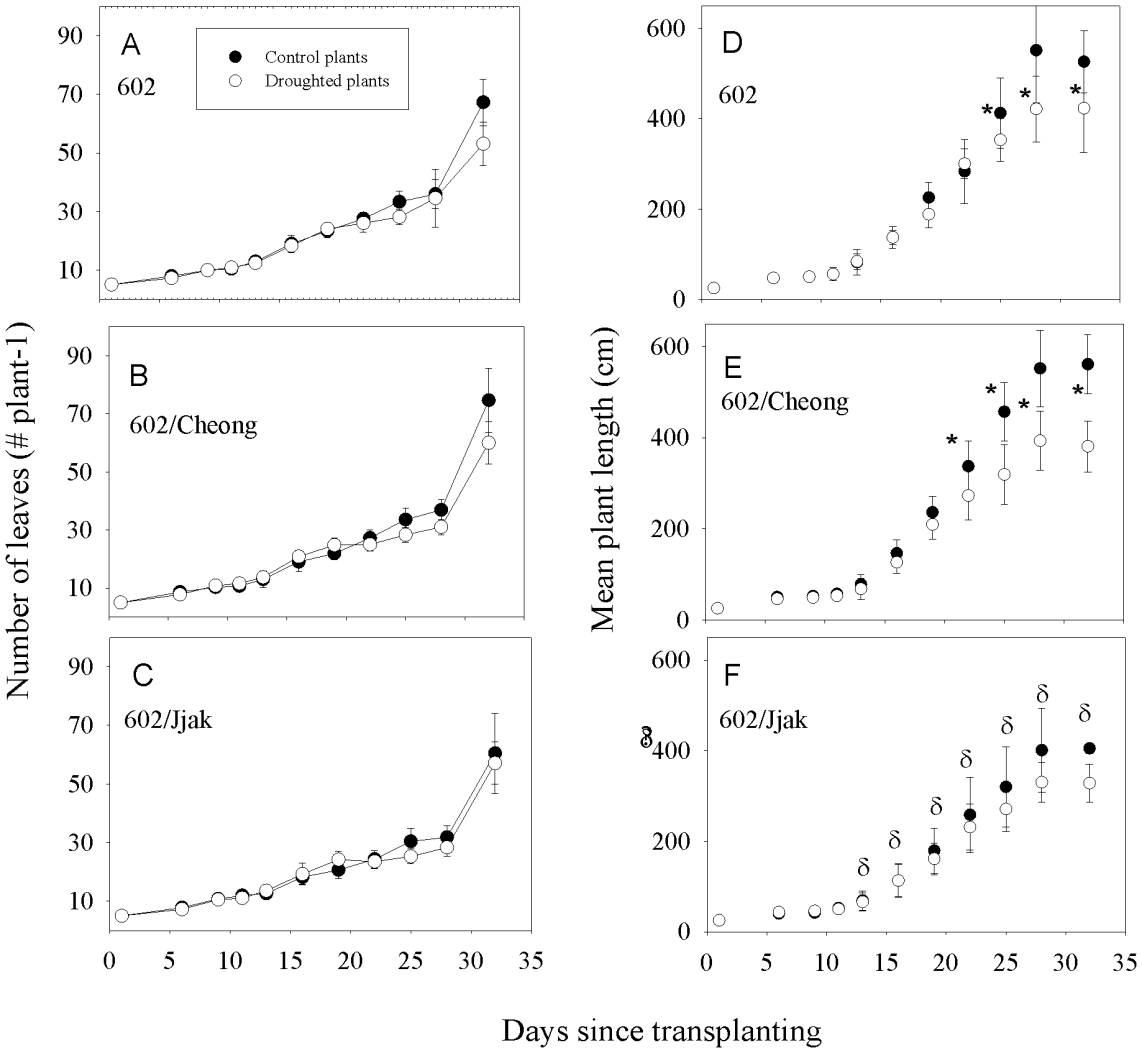
The total number of mature leaves (A, B, C) and the total plant length (D, E, F) for three different lines of tomato grown with daily watering (control) or daily watering until water was withheld between day 20 and day 28 (mild-drought treatment). 602 = ‘BHN 602’; 602/Cheong  =  refers to line ‘BHN 602’ scion grafted onto ‘Cheong Gang’ rootstock; 602/Jjak refers to line ‘BHN 602’ scion grafted onto ‘Jjak Kkung’ rootstock. Error bars represent two standard errors on each side of the mean. *  =  significant difference between control and mild-drought treated plants within a plant type (Student's t-test; p<0.05). δ  =  significant difference between 602/Jjak control plants and 602 control plants (Student's t-test; p<0.05).

The total number of leaves per plant increased steadily from experimental day 0 to day 32 for all three plant-lines (Fig. 2A-C). There were no significant differences in leaf numbers per plant between control and drought-stress treatment plants throughout the experimental period. The total number of leaves for line 602/Jjak was consistently less than the other two plant lines and reached a mean maximum plus and minus two standard errors of 58.9±10.9 while that of line 602 was 67.3±7.9 and that for line 602/Cheong was 74.7±11.1. Total plant length increased logarithmically for the control plants in all three plant-lines from day 0 through day 28 (Fig. 2D-F). After day 28, total plant length leveled off for all plant lines. There was a significant effect of the drought-stress treatment on total plant length from day 19 through day 32 (Table 1). There was a significant effect of plant line on total plant length from day 6 through day 9 and from sample day 16 through day 32 (Table 1). Total plant length of 602/Jjak was significantly less than the other two lines (Student's t-test among plant lines done for each sample date) from day 19–32 (Fig. 2F). The interaction between plant line and drying treatment was not significant over the length of the experiment, although the P value for the interaction decreased consistently from sample day 13 through sample day 32 (Table 1). However, on day 32 the P value for the interaction between plant line and mild drought treatment approached our significance cut-off level.

**Table 1:** Results for 2-way ANOVA performed on total plant length at each sample date.

Sample date	Drought treatment	Plant line	Treatment X line interaction
6	0.664	**0.004**	0.212
9	0.710	**<0.001**	0.133
11	0.328	0.111	0.938
13	0.121	0.232	0.738
16	0.234	**0.031**	0.571
19	**0.004**	**<0.001**	0.507
22	**0.034**	**0.008**	0.393
25	**<0.001**	**<0.001**	0.113
28	**<0.001**	**<0.001**	0.126
32	**<0.001**	**<0.001**	0.056

The effects of plant line, dry treatment and their interaction were tested. Degrees of freedom were: Drought treatment  = 1, Plant line  = 2; dry treatment x plant line interaction  = 2, residuals  = 51, Total = 56. Bold values are significant at the 0.05 level.

During the duration of the experiment there was no loss of tissues (abscission or senescence) for any plant. Total plant weight at final harvest was significantly different between control and mild-drought treatments for both 602 and 602/Cheong, but not for 602/Jjak (Table 2). The mean total weight per plant for the control 602/Jjak plants was similar to that of the mild-drought treated plants for the other two plant lines. A similar result was found for total stem weight, total leaf weight total plant leaf area and the ratio of leaf weight to stem weight (Table 2). There was no significant difference in specific lamina area between control and mild-drought treatment groups for any of the three plant lines. However, there was a trend for mean specific leaf area to be lower in control plants compared with mild-drought treatment plants for all plant lines. The ratio of leaf-lamina area to shoot length was not significantly different between control group and mild-drought treated plants for all three plant-lines (Table 2). Two-way ANOVA indicated a significant effect of plant line and drought treatment for all harvest biomass characteristics (Table 3). The interaction between plant line and drought treatment was only significant for leaf weight and leaf area characteristics at final harvest (Table 3). There was no significant effect of plant line or drought treatment on specific leaf weight or leaf lamina per stem length. However, both plant line and drought treatment had a significant effect on leaf weight per stem weight. The interaction between plant line and drought treatment on leaf weight per stem weight was not significant (Table 3).

**Table 2:** Comparison between three lines of tomato plants treated with either mild-drought or no-drought (control).

	602		602/Cheong		602/Jjak	
Characteristic	control	drought	control	drought	control	drought
Whole plant wt (g)	79.04	45.81	75.03	43.30	50.91	37.97
t-Test p value	**0.002**		**<0.001**		0.092	
Total stem weight (g)	34.21	15.44	28.90	14.89	19.63	12.14
t-Test p value	**0.004**		**0.004**		0.074	
Total leaf weight (g)	44.82	30.37	46.12	28.41	31.29	25.83
t-Test p value	**0.001**		**<0.001**		0.139	
Total leaf area (m^2^)	16.96	12.08	17.36	11.34	11.92	10.33
t-Test p value	**<0.001**		**<0.001**		0.198	
Specific leaf area (cm^2^g^−1^)	383.4	400.9	380.7	405.7	387.5	402.7
t-Test p value	0.416		0.384		0.552	
Leaf/stem wt (g g^−1^)	1.572	1.262	1.690	1.194	1.161	1.097
t-Test p value	**0.007**		**<0.001**		0.391	
Lamina area/stem length (cm^2^ cm^−1^)	32.00	34.30	31.24	30.28	29.66	31.82
t-Test p value	0.250		0.713		0.385	

Student's t-Test assuming unequal variance was used to test for significant differences between mild-drought and control groups for each plant line. P values for two-tailed results are shown. Bold p values indicate significant difference between mild drought and control treatment plants. Significant difference is assigned at the p< = 0.05 level. 602 = BHN 602 non-grafted; 602/Cheong  =  BHN 602 scion grafted onto ‘Cheong Gang’ root stock; 602/Jjak  =  BHN 602 scion grafted onto ‘Jjak Kkung’ root stock.

**Table 3:** Results for 2-way ANOVA performed on total plant weights and leaf area for three different lines of tomato experiencing either mild-drought or no-drought (control).

Characteristic	plant line	drought treatment	interaction
Whole plant weight	**0.003**	**<0.001**	0.125
Total stem weight	**0.017**	**<0.001**	0.249
Total leaf weight	**<0.001**	**<0.001**	**0.036**
Total leaf area	**<0.001**	**<0.001**	**0.022**
Specific leaf area	0.976	0.118	0.935
Leaf wt/stem wt	**0.029**	**<0.001**	0.682
Lamina area/stem length	0.201	0.538	0.659

The effects of plant line, drought treatment and their interaction were tested. Degrees of freedom were: plant line = 2; drought treatment = 1, drought treatment x plant line interaction = 2, residuals = 51, Total = 56. Bold values are significant at the 0.05 level.

**Figure 3:**
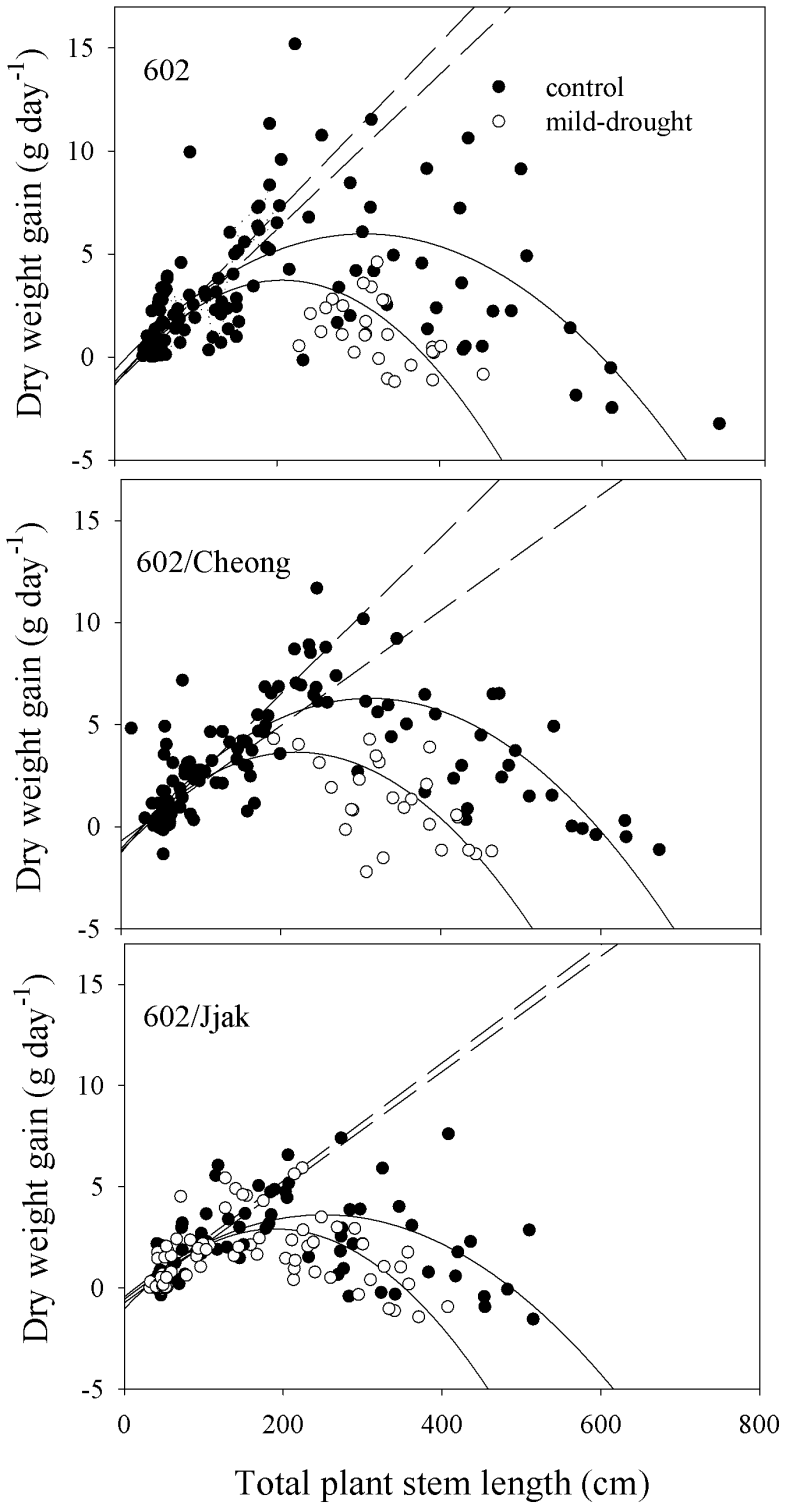
The relationship between growth rate (dry weight gain per day) and total stem length is plotted for three different lines of tomato for mild-drought treated and control plants. 602 = ‘BHN 602’; 602/Cheong  =  refers to line ‘BHN 602’ scion grafted onto ‘Cheong Gang' rootstock; 602/Jjak refers to line ‘BHN 602’ scion grafted onto ‘Jjak Kkung’ rootstock. Dashed lines represent first order regressions for all data below 200 cm length. Solid lines refer to second order regressions for all data.

Before the mild-drought was imposed the relative growth rates (RGR) varied between 0.55 and 0.66 g day^−1^ g^−1^ for both control and mild-drought treatment plant groups (Table 4). After the mild-drought was initiated, RGR varied between 0.09 and 0.27 g day^−1^ g^−1^ for both control and mild-drought treatments (Table 4). Comparisons of RGR before drought initiation with RGR after drought initiation were significant for both the control treatment and the mild-drought treatment (Table 4). Comparisons of RGR of control plants with mild-drought plants were not significant except for non-grafted 602 after the initiation of mild-drought treatment (Table 4). The differential effect of water treatment on growth before and after the mild-drought treatment is best represented by comparing daily weight gain to total plant length (Fig. 3). All plants had a linear increase in daily dry weight gain as stem length increased until a length of approximately 200 cm was reached. Mild-drought treated plant growth departed from their linear relationship with stem length before that of the control plants for all three plant-lines (Fig. 3). Maximum stem length attainable in the pots can be determined by the total stem length that can be attained by the time daily weight gain becomes zero (based on second order regressions in Fig. 3). The greater the number of days for this index, the greater the effect of drought on the plant line. The difference between maximum attainable length for plants in control or mild-drought treatments was 215 cm for line BHN602, 180 cm for line 602/Cheong and 138 cm for line 602/Jjak (Fig. 3).

**Table 4:** The Relative growth rates (RGR) for three lines of tomato experiencing either control conditions or a mild-drought are shown.

	g day^−1^ g^−1^		g day^−1^ g^−1^		P value (Student's t-Test)			
	control		drought		control vs. drought		before vs. after	
Plant Line	before	after	before	after	before	after	control	drought
602	0.56	0.27	0.61	0.09	0.322	**0.025**	**0.006**	**<0.001**
602/Cheong	0.66	.020	0.58	0.14	0.183	0.141	**<0.001**	**<0.001**
602/Jjak	0.57	0.18	0.55	0.14	0.389	0.167	**<0.001**	**<0.001**

Relative growth rate was calculated for each plant line either before or after the drought treatment was initiated. Student's t-Tests are presented for the comparison between the RGR of plants experiencing control or mild-drought treatments either before or after the mild-drought was initiated. Also, Student's t-Tests are presented for the comparison between before and after mild-drought was initiated for both control and mild-drought treatment groups. Significant difference between groups is indicated as a bold face P value. 602 = BHN 602 non-grafted; 602/Cheong = BHN 602 scion grafted onto ‘Cheong Gang’ root stock; 602/Jjak  =  BHN 602 scion grafted onto ‘Jjak Kkung’ root stock.

### Water potential and stomatal conductance

**Figure 4:**
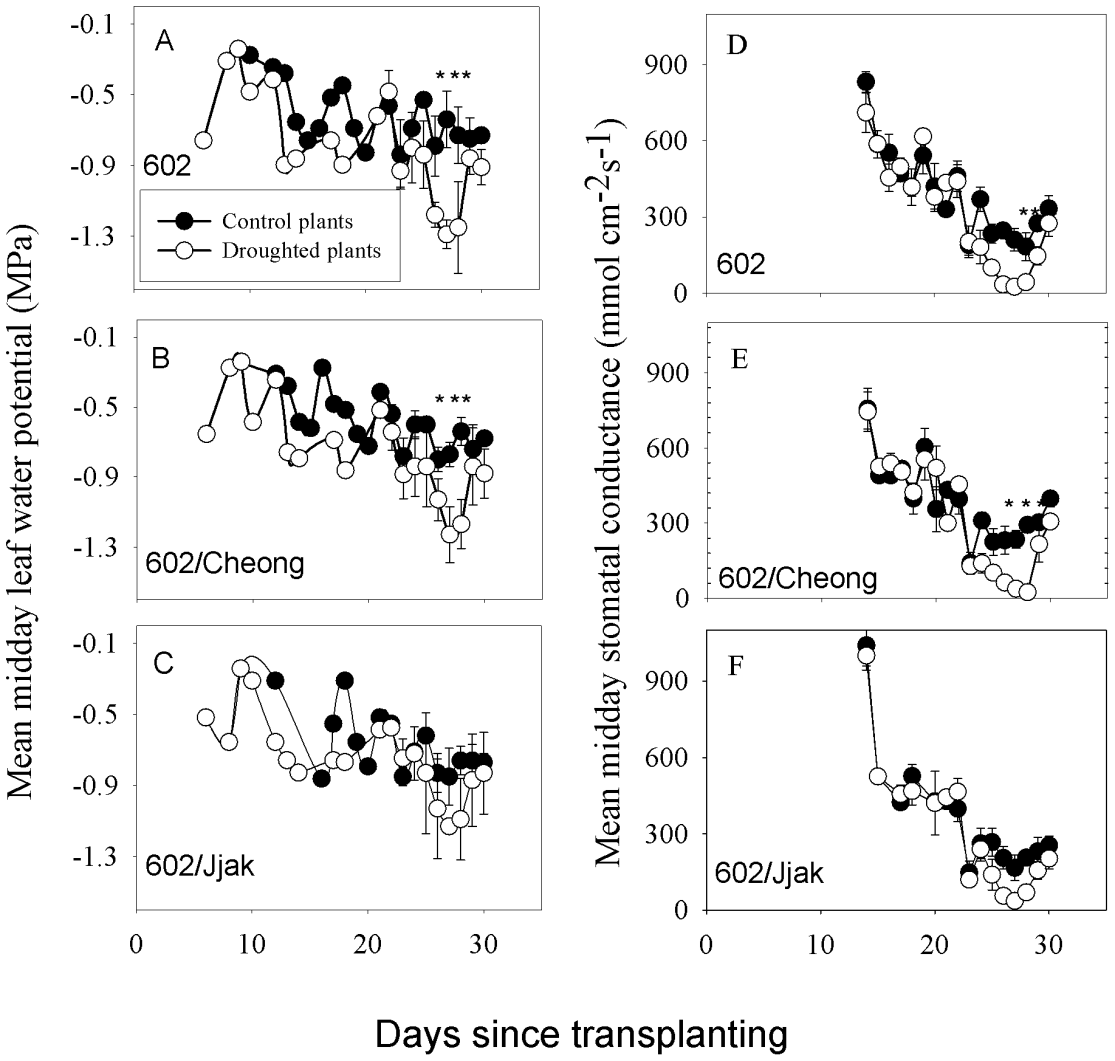
Mean midday water potential (A, B, C) and midday stomatal conductance (D, E, F) for three different lines of tomato grown with daily watering (control) or daily watering until water was withheld between day 20 and day 28 (mild-drought treatment). 602 = ‘BHN 602’; 602/Cheong  =  refers to line ‘BHN 602’ scion grafted onto ‘Cheong Gang’ rootstock; 602/Jjak refers to line ‘BHN 602’ scion grafted onto ‘Jjak Kkung’ rootstock. Error bars represent two standard errors on each side of the mean. *  =  significant difference between control and mild-drought treated plants within a plant type (Student's t-test; p<0.05).

Leaf water potentials, of all three lines, ranged from −0.1 MPa to −0.9 MPa between days 5 and 19 of the experimental period, which was most likely due to variation in PPFD and air temperature among days (Fig. 4A-C). Following day 20, control plants continued to vary within the same leaf water potential range for all three lines. Leaf water potential of mild-drought treatment plants decreased from day 20 until day 29 at which time the plants were re-watered. Line 602 decreased to a mean plus one standard deviation of −1.29±0.08 MPa, line 602/Cheong decreased to −1.23±0.16 MPa and line 602/Jjak decreased to −1.13±0.14 MPa during the water-withholding period. Following re-watering on day 29 mild-drought treatment plants of all three lines returned to the control leaf water potential range. The mean minimum water potential for all lines (using the last two days of mild-drought treatment) were not significantly different from each other (Student's two-tailed t-Test, df = 5, P(T< = t) ranged from 0.4736 to 0.2532).

There was no significant difference in stomatal conductance among plant lines during the pre-treatment period (Fig. 4D-F). Leaf stomatal conductance gradually decreased from over 500 mmol m^−2^ s^−1^ to 400 mmol m^−2^ s^−1^ for all plant lines between day 15 and day 20. On day 20 (the first day of water witholding) midday stomatal conductance decreased for plant line 602 and plant line 602/Cheong to approximately 100 mmol m^−2^ s^−1^ for mild-drought treatment plants. Stomatal conductance for control plants of all lines decreased to 200 mmol m^−2^ s^−1^ because this was a particularly sunny and warm day. Control plant stomatal conductance remained at 200 mmol m^−2^ s^−1^ for the entire mild-drought treatment period for all three plant lines. Stomatal conductance of mild-drought treatment plants decreased from 200 mmol m^−2^ s^−1^ to 10 mmol m^−2^ s^−1^ for all plant lines between day 23 and day 28. Upon re-watering on day 29, stomatal conductance increased for lines 602 and 602/Cheong to over 350 mmol m^−2^ s^−1^; however, stomatal conductance remained at approximately 250 mmol m^−2^ s^−1^ for line 602/Jjak (Fig. 4F).

### Photosynthesis

There was no significant effect of plant line (df = 2, F = 0.137, p = 0.872) on Fv/Fm when all data were combined, but there was a significant effect of mild-drought treatment (df = 1, F =  5.282, p = 0.024). The Fv/Fm for all plants responded to mild-drought the same way because there was no significant effect of the interaction between plant line and mild-drought treatment (df = 2, F = 1.340, p = 0.268) on Fv/Fm when all data were combined. We did detect a decrease in Fv/Fm from a mean of 0.819 in the morning hours (10:00AM–12:00PM) to a mean of 0.798 at midday (1:00PM–2:00PM) and a mean of 0.803 in the afternoon hours (2:00PM–4:00PM) for all plant lines combined. These data indicate significant midday photoinhibition for all plant lines and treatments.

There was no significant effect of plant line on light-saturated photosynthesis (A_max_) when all photosynthetic data were grouped for two-way ANOVA (df = 2, F = 0.613, P = 0.543). There was a significant effect of treatment (control vs. mild-drought) on A_max_ (df = 1, F = 41.19, P = <0.001) and there was no significant interaction between plant line and treatment (df = 2, F = 2.466, P = 0.089). There was no significant effect of plant line on CO_2_-saturated photosynthesis (A_sat_) when all photosynthetic data were combined for two-way ANOVA (df = 2, F = 0.767, P = 0.466). There was a significant effect of treatment (control vs. mild-drought) on A_sat_ (df = 1, F = 43.69, P = <0.001) and there was no significant interaction between plant line and treatment (df = 2, F = 1.522, P = 0.222).

**Figure 5:**
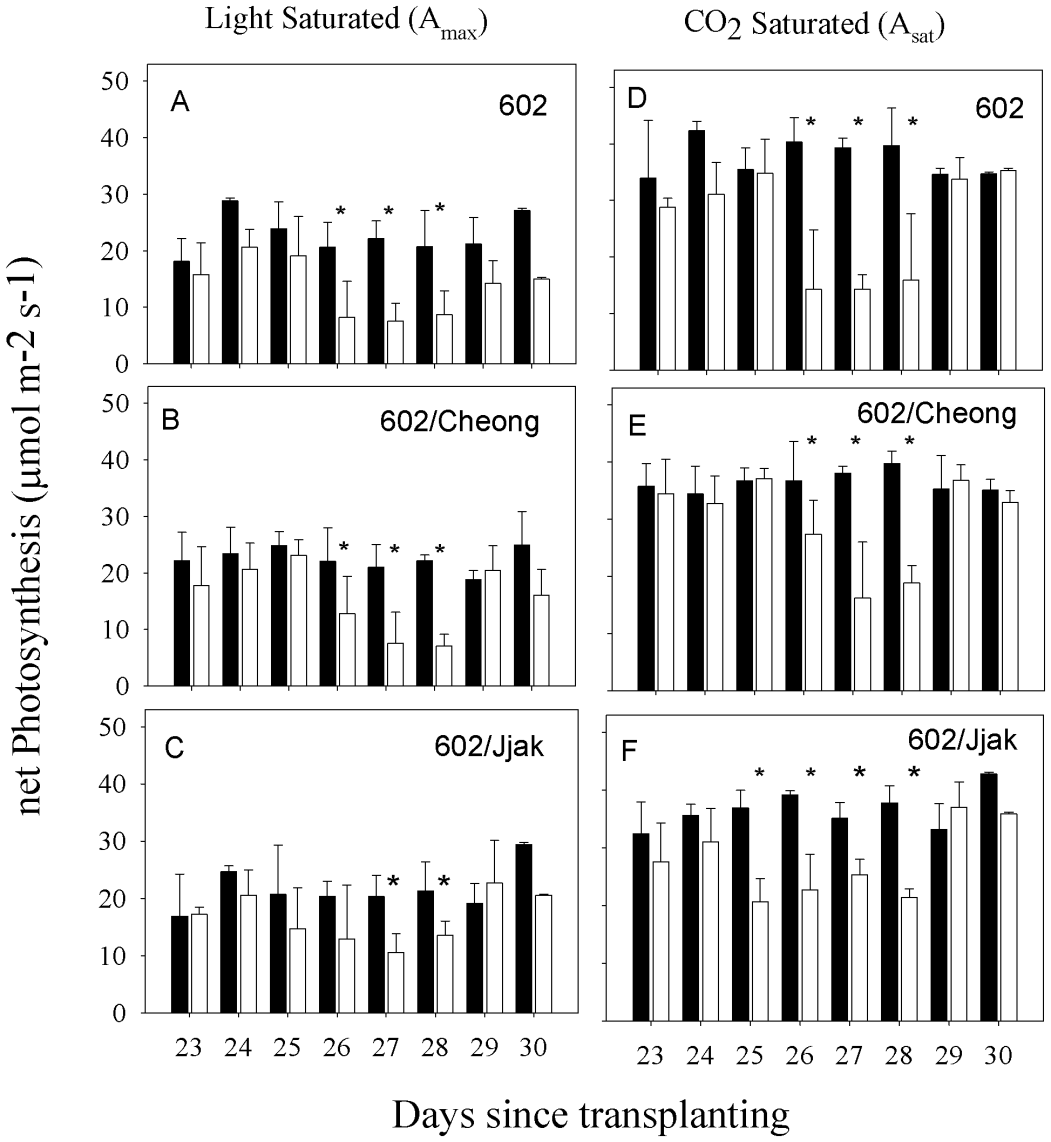
Mean midday light saturated net photosynthesis (A, B, C) and CO_2_ saturated net photosynthesis (D, E. F) for three lines of tomato grown with daily watering (filled bars) or daily watering until water was withheld between day 20 and day 28 (open bars). 602 = ‘BHN 602’; 602/Cheong  =  ‘BHN 602’ scion grafted onto ‘Cheong Gang’ rootstock; 602/Jjak  =  ‘BHN 602’ scion grafted onto ‘Jjak Kkung’ rootstock. Error bars represent two standard errors on each side of the mean. *  =  significant difference between control and mild-drought treated plants within a plant type (Student's t-test; p<0.05).

A_max_ varied from 28.8 to 18.1 µmol m^−2^ s^−1^ and A_sat_ varied from 42.3 to 33.9 µmol m^−2^ s^−1^ for control plants of line 602 (Fig. 5A and D). A_max_ varied from 20.6 to 14.2 µmol m^−2^ s^−1^ and A_sat_ varied from 35.3 to 28.8 µmol m^−2^ s^−1^ for dry treatment plants of line 602 during the times these plants were watered daily (Fig. 5A and D). For days 26–28, during the mild-drought treatment, A_max_ varied from 8.7 to 7.6 µmol m^−2^ s^−1^ and A_sat_ varied from 15.9 to 14.3 µmol m^−2^ s^−1^. Therefore, for line 602, during mild-drought A_sat_ decreased by approximately 52% and A_max_ decreased by approximately 46–58%.

A_max_ varied from 24.9 to 18.8 µmol m^−2^ s^−1^ and A_sat_ varied from 49.8 to 34.4 µmol m^−2^ s^−1^ for control plants of line 602/Cheong (Fig. 5B and E). A_max_ varied from 23.1 to 17.8 µmol m^−2^ s^−1^ and A_sat_ varied from 37.1 to 32.8 µmol m^−2^ s^−1^ for mild-drought treatment plants of line 602/Cheong during the times these plants were watered daily (Fig. 5B and E). For days 26–28, during mild-drought treatment, A_max_ varied from 12.8 to 7.1 µmol m^−2^ s^−1^ and A_sat_ varied from 27.4 to 16.2 µmol m^−2^ s^−1^. Therefore, for line 602/Cheong, during mild-drought, A_sat_ decreased by approximately 26–50% and A_max_ decreased by approximately 41–56%.

A_max_ varied from 29.4 to 16.9 µmol m^−2^ s^−1^ and A_sat_ varied from 42.8 to 32.4 µmol m^−2^ s^−1^ for control plants of line 602/Jjak (Fig. 5C and F). A_max_ varied from 22.7 to 17.4 µmol m^−2^ s^−1^ and A_sat_ varied from 37.0 to 27.6 µmol m^−2^ s^−1^ for dry treatment plants of line 602/Jjak during the times these plants were watered daily (Fig. 5C and F). For days 26–28, during the mild-drought treatment, A_max_ varied from 13.5 to 10.5 µmol m^−2^ s^−1^ and A_sat_ varied from 25.3 to 21.4 µmol m^−2^ s^−1^. Therefore, for line 602/Jjak, during mild-drought, A_sat_ decreased by approximately 23–31% and A_max_ decreased by approximately 38–50%.

**Figure 6:**
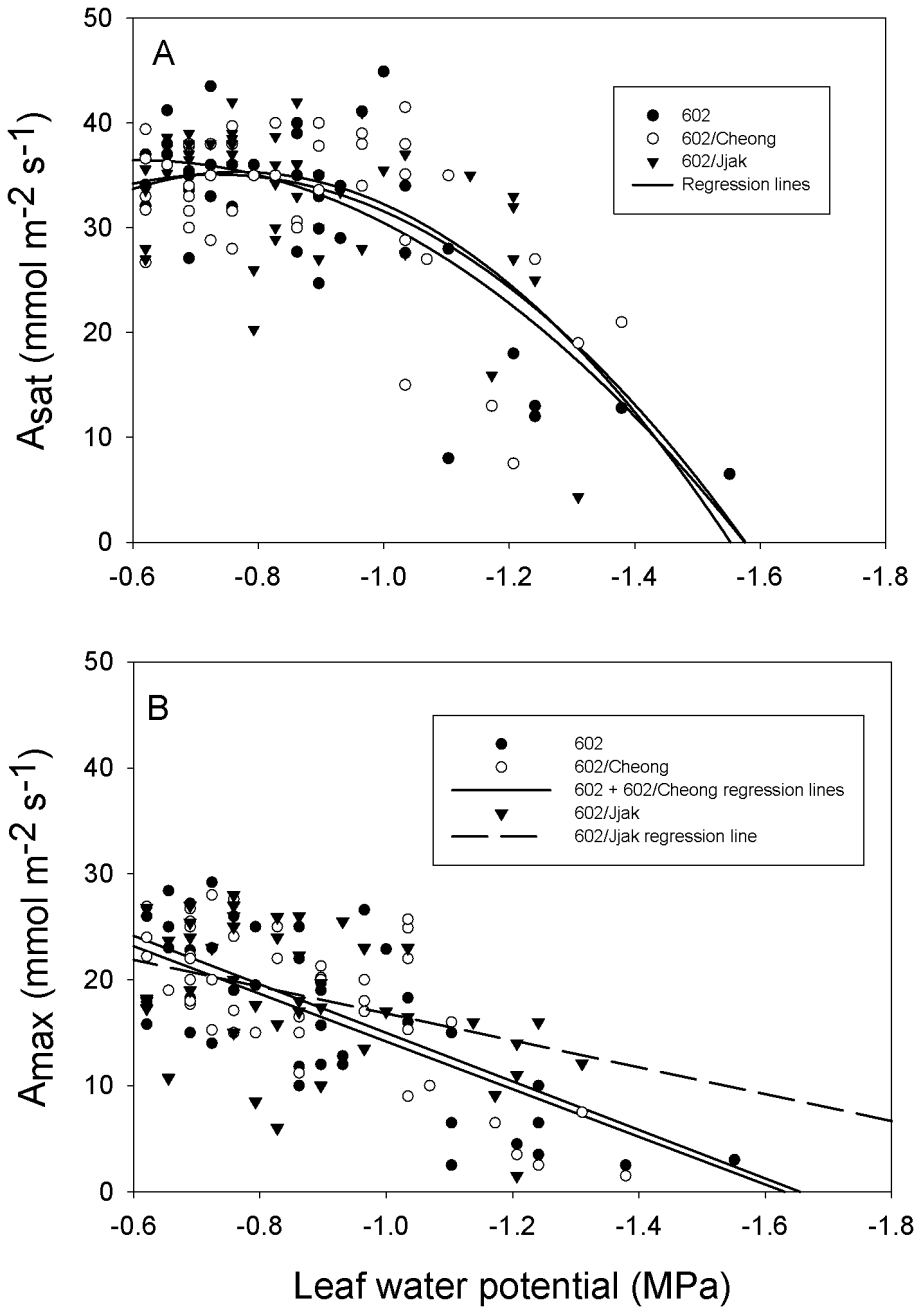
The relationship between mean midday photosynthesis and mean midday leaf water potential for three plant lines of tomato. A) Second order regressions between mean midday CO_2_ saturated photosynthesis (at 1500 µmol m^−2^ s^−1^ PPFD and 1500 ppm CO_2_) and mean midday leaf water potential; B) Linear regressions between mean midday light saturated photosynthesis (at 1500 µmol m^−2^ s^−1^ PPFD and 400 ppm CO_2_) and mean midday leaf water potential. 602 = ‘BHN 602’; 602/Cheong  =  ‘BHN 602’ scion grafted onto ‘Cheong Gang’ rootstock; 602/Jjak  =  ‘BHN 602’ scion grafted onto ‘Jjak Kkung’ rootstock.

A_sat_ remained relatively stable as water potential decreased until −1.0 MPa was reached after which A_sat_ decreased as leaf water potential decreased for all plant lines (Fig. 6). As a result of this biphasic response, the best relationship (maximum R^2^) between A_sat_ and leaf water potential was defined by a second-order, negative-regression. The second-order regressions between A_sat_ and leaf water potential were similar for all plant lines (Fig. 6A). The best relationship (maximum R^2^) between A_max_ and leaf water potential was defined by a negative, first-order, linear-regression for all three-plant lines (Fig. 5B). The slope of the linear regression for lines 602 and 602/Cheong were steeper than that of 602/Jjak (Fig. 6B). However, there was more variation among points for the 602/Jjak line (R^2^ = 0.2) than the other two lines (R^2^ = 0.5).

## Discussion

### Growth effects of grafting

Grafting scions onto rootstock cause the production of callus tissue at the graft junction that may or may not differentiate into xylem and phloem with the same conductance properties as uninterrupted vasculature for transporting materials between the scion and rootstock [9]. As a result, the graft callus could reduce water flow to shoots (decreased hydraulic conductance) and limit photosynthate transport to roots resulting in slower growth than non-grafted controls. In our study, one grafted combination (602/Cheong) grew as well as the control while a different combination (602/Jjak) grew less than the control, although there was no significant difference in plant water potential between the grafted and non-grafted lines. These results suggest that the slower growth of 602/Jjak was unlikely to be caused by a general interference by graft callus. Moreover, our data suggest that different rootstocks have different effectiveness for reducing mild-drought induced growth inhibition.

### Physiological effects of grafting

We tested physiological processes associated with mild-drought growth-inhibition in 602/Jjak compared with the other two plant lines. Our focus was on water relations and photosynthesis traits. We hypothesized that grafting would have no significant effects on water relations and photosynthesis during control growth conditions of mature plants, but that during mild-drought stomatal conductance and photosynthesis would decrease less for 602/Jjak than the other two plant lines. Before the implementation of mild-drought, plant size and variation in daily weather determined leaf water potential and leaf conductance (water flow). Mild drought induced a decrease in midday water potential and a reduction of stomatal conductance for all three plant-lines. However, leaf water potential was higher and stomatal conductance lower during mild-drought for 602/Jjak. Therefore, the low plant leaf area of 602/Jjak resulted in water conservation that could be reflected in irrigation savings when grown in field plantings in comparison with the other two plant-lines. Water conservation would reduce the likelihood that a severe drought would occur until the next rainfall or irrigation.

Mild-drought inhibition of photosynthesis could occur in tomato by a reduction in photosystem II ability to quench photon energy and this would result in a loss of light use efficiency. Photoinhibition is promoted when stomata are closed and intercellular CO_2_ is depleted relative to intercellular O_2_ concentration [21]–[23]. Thus, photoinhibition would be expected to occur in tomato plants experiencing mild-drought, unless non-photochemical quenching can dissipate the excess absorbed photon energy and thereby prevent photoinhibition. Grafted lines of tomato varieties can have higher photosystem II activity under stress conditions than self-grafted and non-grafted controls [24]. We found a significant effect of mild-drought on Fv/Fm for all plant lines. Moreover, we were able to identify a decrease in Fv/Fm between morning hours and afternoon hours for all three plant-lines, which is expected based on known daily patterns in Fv/Fm [25]. Yet, there were no differences in these patterns among the different plant lines. Therefore, photoinhibition of photosystem II during drought was part of the reason why a reduction in A_sat_ occurred for all plant lines in our study, but this loss of light use efficiency during mild-drought was not different among plant lines and could not explain differences in photosynthetic traits or growth among plant lines.

Under light saturated conditions, leaf photosynthesis is dependent on both stomatal and mesophyll conductance. We found clear evidence for inhibition of stomatal conductance during drought. Therefore, stomatal conductance is one main limitation to all three plant-lines during mild-drought. Also, when stomatal limitation to photosynthesis was circumvented by saturating levels of intercellular CO_2_, leaf photosynthesis decreased at water potential less than −1.0 MPa for all plant lines. Therefore, in our experimental system, photosynthesis is primarily limited by stomatal conductance until a water potential of −1.0 MPa is attained. At water potential below -1.O MPa, both mesophyll and stomatal conductance decrease. The decrease in mesophyll conductance during mild-drought was only partially due to a decrease in light use efficiency. In fact, our index on photoinhibition decreased by less than 10% compared with a decrease of 30–50% in A_sat_. This result suggests that during mild drought, nonphotochemical quenching and antioxidant pathways in these tomato lines were adequate to quench most absorbed radiation and detoxify reactive oxygen produced by the fully oxidized photosystem II. This conclusion is supported by the complete recovery of photosynthetic rate in mild-drought treated plants the next morning after re-watering. We noted no evidence of damage to the photochemical reactions by reactive oxygen species produced during mild-drought. Therefore, antioxidant pathways were effective at removing ROS before they could be damaging to photochemical processes. Similar stability of photosystem II quenching ability during stress conditions has been found in other studies using tomato [26], [27]. Mild-drought effects on biochemical processes of photosynthesis must have been a major aspect of mild-drought inhibition because A_sat_ decreased significantly for all plant lines during days 25–28 of the 30 day experiment.

The relationship between leaf water potential and limitation of photosynthesis by mesophyll conductance in BHN602 tomatoes is not significantly affected by grafting, by different rootstocks, or by their interaction. Therefore, there is no significant evidence that a signal molecule or metabolite from the graft or rootstock influenced mesophyll conductance response to mild-drought. However, the % reduction in A_sat_ was smaller for 602/Jjak in comparison to the other lines suggesting that there may be a different response of mesophyll conductance among plant lines that was not found to be significant during mild-drought. This difference may become more accentuated during stronger drought conditions.

There was evidence that stomatal limitation to photosynthesis during mild-drought was affected differently by different rootstocks. For example, the difference between control and mild-drought stomatal conductance was less when Jjak Kkung served as the rootstock in comparison with the Cheong Gang rootstock or non-grafted plants. Also, 602/Jjak plants were able to maintain higher A_max_ compared with 602/Cheong and 602 at low water potential. Thus, 602/Jjak were able to reduce stomatal limitation to photosynthesis at water potential below -1.0MPa (a drought tolerance mechanism) compared to the other grafted lines. These data suggest that 602/Jjak may have been able to maintain a higher guard cell turgor potential during mild-drought than the other two plant lines even though bulk tissue water potential was not significantly different among plant lines. The water conservation of the 602/Jjak combination was due to growth reduction and maintenance of stomatal conductance, which must be due to a rootstock effect because the scions were the same among the two different graft combinations. The different response between the different rootstocks suggests that a root signal from one rootstock (Jjak) is having a greater effect on water conservation than that of the other rootstock (Cheong). Root stock regulation of stomatal conductance by ABA in tomato was previously identified by grafting experiments [28]. ABA signaling can have effects on tomato production during mild-drought [29]. Our overall model for water conservation by the Jjak root stock is a combination of growth reduction (reducing leaf area) and an ability to maintain A_max_ at lower water potentials.

### Implications for agriculture

Particular combinations of scion and rootstock may become an important aspect of future commercial tomato production. We have found evidence that certain scion-rootstock combinations will affect vegetative plant growth, stomatal limitation to photosynthesis, and most importantly, photosynthetic response to mild-drought. In general, we suggest that scion rootstock combinations may have a negative effect on plant growth, but nevertheless, grafting will result in water conservation leading to improved vegetative growth under mild-drought conditions. Water conservation in the plant and soil by rootstock regulation of stomatal conductance can result in less inhibition of growth by mild-drought and potentially less frequent irrigation. Consequently, some specific rootstock-scion combinations could quickly improve water conservation and resistance to mild-drought compared with the time required to develop new water conservative plant lines by conventional breeding or genetic engineering. However, water conservation by growth inhibition demonstrated in this study may have a yield penalty. The balance between the need for water conservation and the potential yield penalty of water conservation must be addressed before particular scion-rootstock combinations are promoted for commercial tomato production. Further phenotypic screening of different rootstock-scion combinations is needed to identify the most water conservative and highest productivity combinations for commercial tomato production.

## Supporting Information

S1 Data Maximum and minimum temperature and relative humidity in the greenhouse over the length of the experiment – Fig. 1.
(XLSX)

S2 Data Greenhouse daily climate information – Fig. 1.
(XLS)

S3 Data Leaf number by date – Text.
(XLS)

S4 Data Shoot length by date – Table 1.
(XLS)

S5 Data Plant length and relative growth rate –Figs. 2 and 3.
(XLS)

S6 Data Final harvest dry weight data file – Tables 2+3.
(XLS)

S7 Data Water potential – Fig. 4.
(XLSX)

S8 Data Fv/Fm and leaf water potential – Fig. 4 and Text.
(XLS)

S9 Data Stomatal conductance – Fig. 4.
(XLSX)

S10 Data Summary of Photosynthesis – Fig. 5.
(XLS)

S11 Data Photosynthesis and water potential – Fig. 6.
(XLS)

## References

[1] Fischer I, Camus-Kulandaivelu L, Allal F, Stephan W (2011) Adaptation to drought in two wild tomato species: the evolution of the *Asr* gene family. New Phytologist 190:1032–1044. 21323928 10.1111/j.1469-8137.2011.03648.x

[2] Boyer JS (1982) Plant productivity and environment. Science 218:443–448. 17808529 10.1126/science.218.4571.443

[3] Bargmann BOR, Laxalt AM, ter Riet B, van Schooten B, Merquiol E, et al (2009) Multiple PLDs required for high salinity and water deficit tolerance in plants. Plant and Cell Physiology 50:78–89. 19017627 10.1093/pcp/pcn173 PMC2638713

[4] Schwarz D, Rouphael Y, Colla G, Venema JH (2010) Grafting as a tool to improve tolerance of vegetables to abiotic stresses: Thermal stress, water stress and organic pollutants. Scientia Horticulturae 127:162–171.

[5] Sánchez-Rodríguez E, Rubio-Wilhelmi MD, Rios JJ, Blasco B, Rosales MA, et al (2011) Ammonia production and assimilation: Its importance as a tolerance mechanism during moderate water deficit in tomato plants. Journal of Plant Physiology 168:816–823. 21316797 10.1016/j.jplph.2010.11.018

[6] Choi JY, Seo YS, Kim SJ, Kim WT, Shin JS (2011) Constitutive expression of *CaXTH3*, a hot pepper xyloglucan endotransglucosylase/hydrolase, enhanced tolerance to salt and drought stresses without phenotypic defects in tomato plants (*Solanum lycopersicum* cv. Dotaerang). Plant Cell Reports 30:867–877. 21207033 10.1007/s00299-010-0989-3

[7] Ziaf K, Loukehaich R, Gong PJ, Liu H, Han QQ, et al (2011) A multiple stress-responsive gene *ERD15* from Solanum pennellii confers stress tolerance in tobacco. Plant and Cell Physiology 52:1055–1067. 21576192 10.1093/pcp/pcr057

[8] Gong PJ, Zhang JH, Li HX, Yang CX, Zhang CJ, et al (2010) Transcriptional profiles of drought-responsive genes in modulating transcription signal transduction, and biochemical pathways in tomato. Journal of Experimental Botany 61:3563–3575. 20643807 10.1093/jxb/erq167 PMC2921197

[9] Martínez-Ballesta MC, Alcaraz-López C, Muries B, Mota-Cadenas C, Carvajal M (2010) Physiological aspects of rootstock-scion interactions. Scientia Horticulturae 127:112–118.

[10] Lee JM (2003) Advances in vegetable grafting. Chronicles of Horticulture 43:13–19.

[11] Sharma S, Sharma N (2008) Rootstocks affect growth, water relations, gas exchange, and anatomy of ‘Flemish Beauty’ pear under water stress. Journal of Horticultural Science & Biotechnology 83:658–662.

[12] Krishnamurthy KS, Rerna J, Mathew PA, Krishnamoorthy B (2008) Identification of suitable *Myristica* species/related taxa as rootstock to combat drought in nutmeg. Indian Journal of Horticulture 65:204–208.

[13] Isaakidis A, Sotiropoulos T, Almaliotis D, Therios I, Stylianidis D (2004) Response to severe water stress of the almond (*Prunus amygdalus*) ‘Ferragnes’ grafted on eight rootstocks. New Zealand Journal of Crop and Horticultural Science 32:355–362.

[14] Tuwei G, Kaptich FKK, Langat MC, Smith BG, Corley RHV (2008) Effects of grafting on tea 2. Drought tolerance. Experimental Agriculture 44:537–546.

[15] Silva VA, Antunes WC, Salgado Guimãraes BL, Cardoso Paiva RM, Silva VdF, et al (2010) Physiological response of Conilon coffee clone sensitive to drought grafted onto tolerant rootstock. Pesquisa Agropecuária Brasileira 45:457–464.

[16] Pire R, Pereira A, Diez J, Fereres E (2010) Influence of rootstock and irrigation level on water relations of grapevines grown under tropical conditions. Journal of Food Agriculture & Environment 8:703–709.

[17] Sommer KJ, Hancock F, Downey MO (2010) Resilience of Sultana (*Vitis vinifera*) to drought and subsequent recovery: field evaluation of nine rootstock scion combinations. South African Journal of Enology and Viticulture 31:181–185.

[18] Rivard CL, Louws FJ, Peet MM, O'Connell S (2008) High tunnels and grafting for disease management in organic tomato production. Phytopathology 98:S133–S133.

[19] Bausher MG (2011) Grafting technique to eliminate rootstock suckering of grafted tomatoes. Hortscience 46:596–598.

[20] Rivard CL, Louws FJ (2008) Grafting to manage soilborne diseases in heirloom tomato production. Hortscience 43:2104–2111.

[21] Demmig-Adams B, Adams WW III (1992) Photoprotection and other responses of plants to high light stress. Annual Review of Plant Physiology and Plant Molecular Biology 43:599–626.

[22] Cornic G, Ghashghaie J, Genty B, Briantais JM (1992) Leaf photosynthesis is resistant to mild drought stress. Photosynthetica 27:295–309.

[23] Xie TT, Su PX, Shan LS (2010) Photosynthetic characteristics and water use efficency of sweet sorghum under different watering regimes. Pakistan Journal of Botany 42:3981–3994.

[24] He Y, Zhu ZJ, Yang J, Ni XL, Zhu B (2009) Grafting increases the salt tolerance of tomato by improvement of photosynthesis and enhancement of antioxidant enzymes activity. Environmental and Experimental Botany 66:270–278.

[25] Liu YB, Zhang TG, Li XR, Wang G (2007) Protective mechanism of desiccation tolerance in *Reaumuria soongorica*: leaf abscission and sucrose accumulation in the stem. Science in China Series C-Life Sciences 50:15–21. 10.1007/s11427-007-0002-8 17393078

[26] Albacete A, Martinez-Andujar C, Ghanem ME, Acosta M, Sanchez-Bravo J, et al (2009) Rootstock-mediated changes in xylem ionic and hormonal status are correlated with delayed leaf senescence, and increased leaf area and crop productivity in salinized tomato. Plant Cell and Environment 32:928–938. 10.1111/j.1365-3040.2009.01973.x 19302168

[27] Erzin V, Pena Rdl, Ahanachede A (2010) Physiological and agronomic criteria for screening tomato genotypes for tolerance to salinity. Electronic Journal of Environmental, Agricultural and Food Chemistry 9:1641–1656.

[28] Holbrook NM, Shashidhar VR, James RA, Munns R (2002) Stomatal control in tomato with ABA-deficient roots: response of grafted plants to soil drying. Journal of Experimental Botany 53:1503–1514. 12021298

[29] Jensen CR, Battilani A, Plauborg F, Psarras G, Chartzoulakis K, et al (2010) Deficit irrigation based on drought tolerance and root signalling in potatoes and tomatoes. Agricultural Water Management 98:403–413.

